# Effectiveness of Combined Preoperative Sublingual Misoprostol and Intravenous Tranexamic Acid on Intraoperative Blood Loss During Elective Caesarean Section: A Randomised, Blinded, Placebo-Controlled Trial

**DOI:** 10.7759/cureus.41041

**Published:** 2023-06-27

**Authors:** Ubong B Akpan, Obinna Ugwuoke, Edet Ekpo, Ezukwa Omoronyia, John Ekabua

**Affiliations:** 1 Department of Obstetrics and Gynaecology, University of Calabar Teaching Hospital, Calabar, NGA; 2 Department of Obstetrics and Gynaecology, Nigerian Airforce Medical Centre, Calabar, NGA; 3 Department of Obstetrics and Gynaecology, Universty of Calabar Teaching Hospital, Calabar, NGA

**Keywords:** intraoperative blood loss, tranexamic acid, postpartum haemorrhage, misoprostol, hematocrit value, elective surgery, caesarean section

## Abstract

Introduction: The objective of this study was to evaluate the effect of preoperative administration of sublingual misoprostol and intravenous tranexamic acid (TXA) on intraoperative blood loss during elective caesarean sections.

Methods: This was a double-blinded, randomised, placebo-controlled study involving 116 women scheduled for elective caesarean sections. The treatment arm, group 1 (n=58), received 1000 mg of intravenous tranexamic acid 10-15 minutes before skin incision and 600 mcg of sublingual misoprostol after sub-arachnoid anaesthesia and before skin incision. Group 2 (n=58) received placebos; both groups had oxytocin injections at the delivery of the placenta. The data were analyzed using IBM® Statistical Package for the Social Sciences (SPSS) version 24 (IBM Corp., Armonk, NY).

Results: The primary outcome was the intraoperative blood loss and the difference between preoperative and postoperative hematocrit values in both groups. The mean intraoperative blood loss was significantly lower in the study group than in the control group (308.552 ± 42.991 mL versus 736.414 ± 171.889 mL, p<0.001). The differences between the preoperative and post-operative hematocrit values were also significantly lower in the study group than in the control group (2.212% ± 0.805% versus 5.660% ± 2.496%, p<0.001).

Conclusion: Preoperative administration of 1000 mg of intravenous tranexamic acid and 600 mcg of sublingual misoprostol significantly reduced blood loss related to elective caesarean delivery.

## Introduction

Annually, about 14 million women suffer from postpartum haemorrhage (PPH), and 2% of deaths that follow it often occur two to four hours from the onset of bleeding [1]. Postpartum haemorrhage is responsible for 25% to 30% of maternal deaths globally [2,3]. For every maternal death, there are many more that are left with a life-changing disability to live with [1]. Most of these maternal deaths occurring as a result of postpartum haemorrhage could be prevented by the use of medications such as uterotonics and antifibrinolytic agents [4].

A caesarean section is a lifesaving obstetric operation that may be necessary and sometimes the only feasible option of delivery in high-risk pregnancies. However, caesarean sections are not without complications. Caesarean section is associated with twice the blood loss associated with vaginal delivery and, when in excess of 1000 mL, is termed PPH [5]. The risk of blood loss increases with longer operating times, poor surgical technique, repeat caesarean sections, preterm caesarean sections, and elective caesarean sections because the lower uterine segment is poorly formed and the uterus has not started contracting [6,7]. Also, women with placenta previa, morbidly adherent placenta, multiple pregnancies, fibroid in pregnancy, pre-eclampsia, prolonged labour, and abruptio placenta are at higher risk of severe bleeding during caesarean section [8].

Furthermore, the risk of uterine atony following a caesarean section is higher compared to vaginal delivery [9]. Postpartum anaemia is associated with poor wound healing, prolonged hospital stays, puerperal sepsis, depression, poor lactation, blood transfusions, reduced quality of life, impaired cognition, and emotional instability [6].

Therefore, in order to reduce the risk of these complications, feasible preventive measures to reduce blood loss during caesarean sections should be instituted. Thus, controlling blood loss during a caesarean section will have a significant impact on maternal mortality and morbidity following a caesarean section and thus make this life-saving procedure safer. Globally, oxytocin is routinely used to prevent excessive uterine bleeding during caesarean sections. However, despite its effectiveness, 10-40% of women need additional uterotonic therapy such as methyl ergometrine or 15-methyl prostaglandin F2α to prevent uterine atony [10]. In addition, oxytocin needs to be stored between 4 and 8 degrees Celsius to optimise its effectiveness [10]. This may be difficult to achieve in most middle- and low-income countries, where power outages are a formidable challenge.

Misoprostol and pro-hemostatic agents such as tranexamic acid (TXA) provide a complementary biochemical hemostatic effect on the uterus. These drugs have been extensively studied individually for the prevention and treatment of postpartum haemorrhage following vaginal delivery [11-13]. However, data on the combined use of both agents during caesarean delivery are lacking; hence, this research seeks to prove the superiority or otherwise of this regimen with regards to intraoperative blood loss during caesarean section.

## Materials and methods

Study setting

The study was conducted at the Obstetrics and Gynaecology Department of the University of Calabar Teaching Hospital. The hospital serves as a training institution for undergraduate students and resident postgraduate doctors.

The hospital is in the Calabar metropolis, the Cross River state capital, in the South-South region of Nigeria. Two other major health institutions, General Hospital Calabar and the Nigerian Navy Reference Hospital, are also situated in the Calabar metropolis. The hospital serves as a major referral centre for both government-owned and privately owned hospitals in Cross River State. About an average of 2000 deliveries take place in the hospital per year.

Study population

The study population was pregnant women with singleton pregnancy at term who were admitted for delivery by elective caesarean section. A total of 2277 deliveries took place at the hospital, according to the review of records from January 1, 2019 to December 31, 2019. Nine hundred and sixty-nine (969) of the deliveries were via caesarean section, giving a caesarean section rate of 42.56%. 572 of these caesarean sections were performed as elective cases, giving a rate of 59.02%.

Study design

It was a double-blinded, randomised clinical study that compared the effect of preoperative administration of sublingual 600 mcg of misoprostol and 1000 mg of intravenous tranexamic acid with a placebo. The primary outcome of the study was to assess the intraoperative blood loss in both groups and the perioperative fall in haemoglobin concentration. The secondary outcome was to assess the incidence of postpartum haemorrhage, the need to use additional uterotonic drugs, the need for further surgical interventions, and the need for blood transfusions.

Inclusion Criteria

Participants of the study consisted of all pregnant women with singleton pregnancy at term who were admitted for elective caesarean section with gestational ages in the range of 37-42 weeks, women within the age range of 18-40 years who consented to take part in the study, and women who have spinal anaesthesia for elective caesarean section.

Exclusion Criteria

Pregnant women with the following conditions were excluded from the study: women with underlying disease (heart, liver, kidney, pulmonary, haemoglobinopathies, etc.); allergies to misoprostol and or tranexamic acid (such as a known allergy or thromboembolic event during pregnancy); abnormal placentation (placental abruptio, placenta previa, etc.); history of greater than two caesarean sections; history of classical caesarean sections; previous history of uterine rupture, preterm delivery, eclampsia, concurrent anticoagulant therapy, concurrent long-term use of steroids; and dissatisfaction to continue participation in the study. Participants with incomplete data or inadequate information were also excluded from the study.

Sampling procedure

The patients who presented to the antenatal and labour wards for elective caesarean sections were used as the sampling frame. Using the computer-generated codes by simple randomization, the patients were recruited into group 1 (the treatment arm) or group 2 (the placebo arm) of the study if they met the inclusion criteria and gave consent for the study. The patient’s biodata, obstetric history, indication for the index caesarean section, and any associated co-morbidities were noted. Furthermore, their routine laboratory investigations, with particular emphasis on the haemoglobin, platelet count, and coagulation profile where applicable, were also reviewed. Obstetric ultrasound scan reports from at least 36 weeks of gestational age were reviewed, documenting placental localization and the number of foetuses.

Research Protocols

A 10 mL syringe containing clear fluid (injection tranexamic acid 1000 mg or 10 mL of injection water) and a drug envelope containing three tablets (tablet misoprostol 600 mcg or tablet placebo) were retrieved from a pharmacist by a trained assistant who was unaware of their contents. Depending on the computer-generated code for a client, the pharmacist filled the syringe with either a placebo or 1000 mg of tranexamic acid and inserted three tablets of misoprostol (200 mcg each)/placebo into the drug envelope. The drugs/placebos were administered as described before the commencement of surgery. All patients had five millilitres of venous blood taken while securing an intravenous line in the theatre for preoperative haemoglobin estimation before any intervention. Patients assigned to group 1 (n=58) received 1000 mg of tranexamic acid in 100 mL of normal saline over 10 minutes before spinal anaesthesia and sublingual misoprostol (600 mcg, misoclear) immediately after spinal anaesthesia and before skin incision. Patients assigned to group 2 (n=58) received the placebo (Injection water and placebo tablets) in a similar manner. The theatre team and the patients were blinded to the contents of each syringe and envelope.

**Figure 1:**
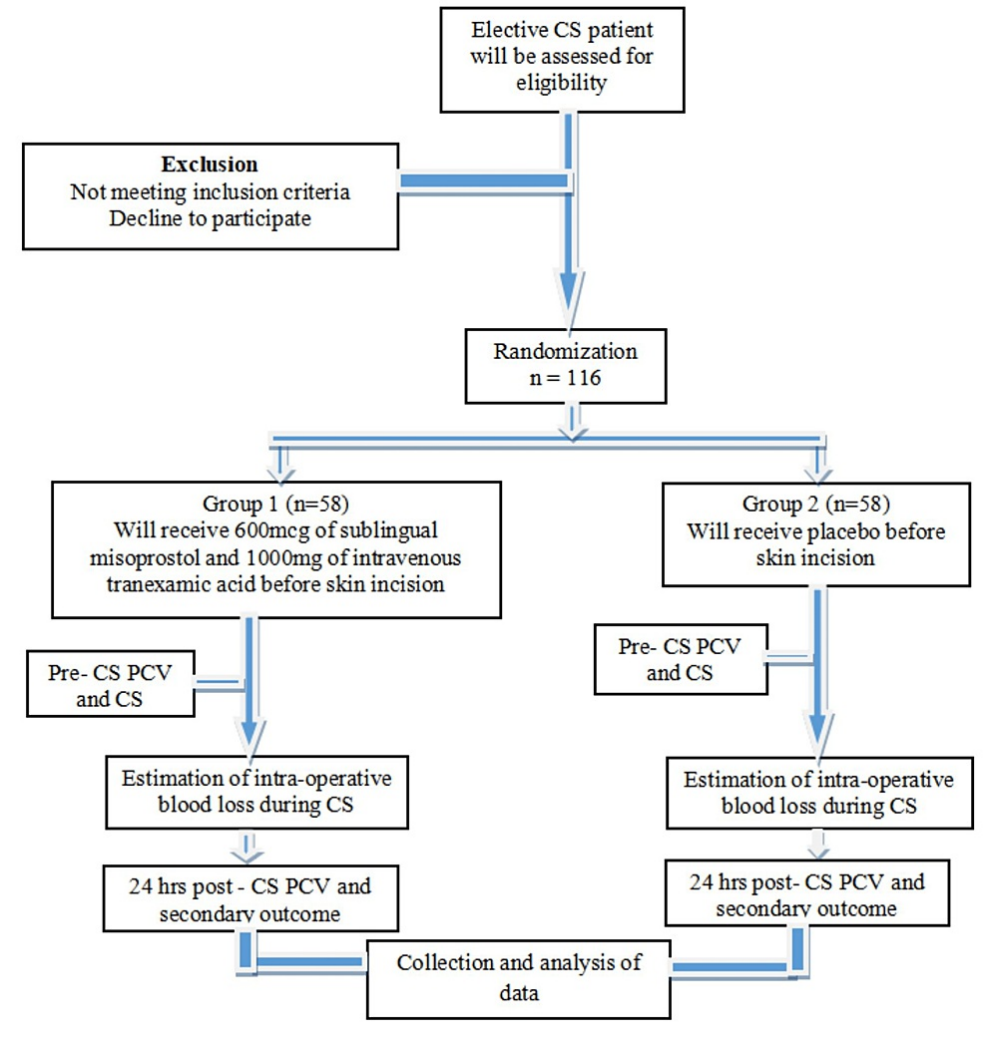
Flow chart summarizing the research protocol CS: ceasarean section, PCV: packed cell volume

The caesarean section technique was the same for all the recruited women and was done under spinal anaesthesia. The time interval between the drug (misoprostol, tranexamic acid, or placebo) administration and delivery of the foetus was noted in both groups and recorded in the data collection form. Additional uterotonic was requested by the surgeon during the surgery on clinical grounds occasionally, and this was indicated as additional uterotonic during data analysis (Figure 1). Also, packed cell volume was reassessed 24 hours after surgery by the Wintrobe centrifugation method.

Estimation of Blood Loss

Assessment of blood loss was commenced after skin incision by the gravimetric method. During the operation, an isolated suction was used for the evacuation of amniotic fluid through a small incision over the lower uterine segment, and a separate suction was used for the collection of blood after the delivery of the baby and placenta. The surgery assistant was responsible for the collection of blood and amniotic fluid in two separate suction sets, and the scrub nurse was responsible for weighing surgical swabs and linen before the surgical swabs were weighed with their package covering, after which they were exposed and dropped on the surgical tray. Thereafter, the package was weighed alone, and the difference in weight was the weight of the surgical swabs before and after the operation. Fixed-sized mops of 20 cm × 10 cm were used in the study. A weight difference of 1 g was equivalent to 1 mL of blood [14]. The collected blood in the suction bottle was added to the blood from the swabs and clots. A highly accurate digital weighing balance was used for weighing the mops and swabs to get the perioperative increase in their weights.

During the study, preoperative and 24-hour postoperative packed cell volumes were estimated as an additional method of assessing blood loss during surgery and 24 hours postoperatively.

Data analysis

Data were collected and statistically analyzed using IBM SPSS Statistics for Windows, Version 24.0 (IBM Corp., Armonk, NY). Quantitative variables were presented in terms of mean ± standard deviation, and qualitative variables were expressed as frequency and percentage. An independent t-test was used to compare the mean of the scale variable, while Pearson’s Chi-square test was used to test for statistical significance in the categorical variable. An analysis of the effect of the duration of surgery and the incision-to-delivery interval of surgery on intraoperative blood loss was done using multiple linear regression. A P-value of less than 0.05 was considered significant.

Ethical consideration

Participation in this research was voluntary, and the principle of confidentiality was strictly adhered to. Each subject was duly counselled, and a prepared consent form was signed. The women who did not consent to participate in the study were in no way discriminated against with regard to medical treatment for their conditions. Also, formal approval for the study was obtained from the health research and ethics committee. Women who had PPH or other complications were managed promptly using existing hospital protocols.

## Results

A total of 116 women (58 in each group) were included in the final analysis. The mean age of the participants was 31.086 ± 4.469 and 30.552 ± 4.791 years for group 1 (treatment arm) and group 2 (placebo arm), respectively. The baseline demographic characteristics of both groups were similar, with no statistically significant differences. The mean birth weight of their infants was 3.379 ± 0.414 and 3.388 ± 0.524 kg in the treatment arm and the placebo arm, respectively, and there was no statistically significant difference in the two groups (P=0.199). Their demographic details are shown in Table 1.

**Table 1:** Demographic details BMI: body mass index, GA: gestational age

Variable	Group 1 (misoprostol + tranexamic acid)	Group 2 (placebo)	P-value
	n=58	n=58	
Mean age in years	31.086 ± 4.469	30.552 ± 4.791	0.673
Mean GA in weeks	38.879 ± 1.557	38.897 ± 1.597	0.677
BMI (kg/M^2^)	33.429 ± 3.570	34.760 ± 4.963	0.289
Parity			
Primiparous: n (%)	42 (36.2%)	36 (31%)	0.547
Multiparous: n (%)	16 (13.8%)	22 (19.0%)	

In assessing the impact of combined treatment with preoperative misoprostol and TXA, the study showed that women in the treatment arm had a significant decrease in blood loss during caesarean section compared with the women in the placebo arm. Also, the differences in hematocrit values were statistically significant, as seen in Table 2. The treatment arm had a significantly lower risk for postpartum haemorrhage, blood transfusion, and the need for additional uterotonic medication during caesarean delivery (Table 3).

**Table 2:** Comparing the intraoperative blood loss and peri-operative hematocrit values P-value (p < 0.05)* = statistical significant.

Variable	Group 1 (misoprostol + tranexamic acid)	Group 2 (placebo)	P-value
	n=58	n=58	
Intraoperative blood loss	308.552 ± 42.991	736.414 ± 171.889	0.001*
Preoperative hematocrit	32.655 ± 2.268	32.757 ± 3.337	0.072
Postoperative hematocrit	30.443 ± 2.517	27.097 ± 4.392	0.001*
Hematocrit difference	2.212 ± 0.805	5.660 ± 2.496	0.001*

**Table 3:** The secondary outcomes *Significant P-value, CI: confidence interval, PPH: postpartum haemorrhage, RR: relative risk.

Outcome	Frequency (%)	P-value	RR	95%CI	
				Lower	Upper
Additional uterotonics					
Treatment group (n=58)	1 (1.76)	0.001*	0.173	0.052	0.581
Placebo group (n=58)	14 (12.1)				
PPH					
Treatment group (n=58)	1 (0.9)	0.001*	0.061	0.008	0.482
Placebo group (n=58)	13 (11.2)				
Blood transfusion					
Treatment group (n=58)	1 (0.9)	0.0001*	0.216	0.038	1.214
Placebo group (n=58)	6 (5.3)				

The regression analysis showed the effect of the duration of surgery and the ‘incision to baby extraction’ time on intraoperative blood loss. Thus, as the duration of surgery increases, the estimated blood loss (EBL) increases. In 19.1%, the increase in intraoperative blood loss was accounted for by the relatively longer duration of surgery (adjusted r^2^= 0.191, P < 0.001). In addition, the unstandardized coefficient - B, showed that for every minute increase in the duration of surgery, there was a 4.6 mL increase in intraoperative blood loss (B=4.568, P<0.001).

Furthermore, correlation analysis revealed a weak positive relationship between intraoperative blood loss and duration of surgery (r=0.194) in the treatment arm, but this relationship was not significant in the treatment group (P=0.144). On the contrary, the duration of surgery had a stronger positive correlation with the EBL (r=0.461), and this relationship was significant (P<0.05) in the placebo group. For every minute increase in the duration of surgery, the intraoperative blood loss increased by 4.6 mL. This implies that the effect of the treatment with misoprostol and tranexamic acid induces strong contractions of the uterus and reduces the amount of blood loss as the surgery progresses.

## Discussion

Caesarean sections are associated with a higher risk of postpartum haemorrhage. In this study, the impact of misoprostol and tranexamic acid administered preoperatively significantly reduced intraoperative blood loss. This is in keeping with previous reports, which showed the effects of the preoperative administration of these agents on blood loss during caesarean delivery [15-20]. In comparing the mean blood loss in our study with one of the ‘misoprostol only’ studies, we found that the mean blood loss in these women who received a combination of misoprostol and TXA was 308.55±42.99 mL compared to the mean of 595±108 mL in women treated with misoprostol alone [21]. This difference is highly significant considering the average blood volume of a pregnant woman, and it suggests the potential benefit of this combination treatment on overall maternal health.

Furthermore, when we compared our findings with one of the ‘TXA only’ studies in North Africa, the mean intraoperative blood loss in that study was much higher than that of our study (583.23±379.52 versus 308.55±42.99) [22]. This difference of more than 250 mL further shows the superiority of combining misoprostol and TXA treatment in preventing PPH in high-risk caesarean deliveries.

The timing and route of administration of misoprostol are important with regard to bioavailability, efficacy, and side effects. The sublingual route ensures fast absorption, less effect of first-pass metabolism, and fewer alimentary side effects. Therefore, the administration of these drugs prior to skin incision in this study likely contributed to a marked reduction in intraoperative blood loss as compared to the study by Bose et al., where the drugs were administered at cord clamping [23].

The mean postoperative hematocrit was higher for the Misoprostol + TXA arm than that of the placebo arm (30.443 ± 2.517% versus 27.097 ± 4.392%, p=0.001). This is in keeping with the findings from other studies [24-26]. Thus, a combination of preoperative misoprostol and TXA reduces the risk of postpartum anaemia following caesarean delivery. Women with low postpartum packed cell volumes are at higher risk for puerperal sepsis, anaemic morbidity, surgical wound infection, and prolonged hospital stays.

Furthermore, the mean change in hematocrit was lower for the treatment arm than for the placebo arm (2.212 ± 0.805% versus 5.660% ± 2.496% p<0.001). The findings from this study were also similar to those of Youssef et al. [27] and Sood et al. [21], in which the perioperative decrease in hematocrit value was significantly less in the misoprostol group (2.61 ± 0.87 vs. 3.03 ± 0.76%, p = 0.0018). Also, the study from Kumar et al. [21] showed a lesser fall in postoperative hematocrit value for preoperative misoprostol when compared to postoperative misoprostol, similar to this study. This mean difference was, however, higher than that of this study (4.305±2.26% versus 2.212±0.805%). This could be explained by the higher dose of misoprostol, the route of administration (sublingual), and the combination of misoprostol with the tranexamic acid regimen in this study. Therefore, the use of the sublingual route for the administration of misoprostol preoperatively in addition to tranexamic acid administered intravenously seems likely to give an added advantage over the rectal route alone.

Also, this study showed that the proportion of women requiring additional uterotonics was significantly higher in the placebo arm than in the misoprostol-TXA arm, which is in keeping with the study of Youssef et al. The lesser number of participants requiring additional oxytocin in this study compared to the Youssef study (1.7% versus 7%) may be attributed to the possible impact of combined misoprostol-TXA [27]. Also, in a study by Sood et al. [21], the need for additional uterotonic agents was significantly less in the misoprostol group compared to the placebo group (22.2% versus 42.8%). The higher percentage of women in the misoprostol group in that study who had additional uterotonic as compared to our study could be due to the relatively higher dose of misoprostol used in our study. Thus, we recommend 600 mcg of misoprostol as PPH prophylaxis in high-risk women undergoing caesarean sections. When available, 1000 mg of TXA should be added. The recommended dose of misoprostol for prophylaxis against PPH is 400 mcg to 600 mcg.

Furthermore, the need for blood transfusion in this study was significantly lower for the treatment arm when compared with the placebo arm (0.9% versus 11.2% of participants). This is also in keeping with the findings from previous research [28,29]. Additionally, the incidence of postpartum haemorrhage in this study was significantly lower in the misoprostol-TXA arm than in the placebo arm (0.9% versus 5.4%). Thus, the use of preoperative sublingual misoprostol and intravenous tranexamic acid could reduce the incidence of postpartum haemorrhage and the need for blood transfusion during and after caesarean section.

In considering the relative effect of intraoperative time as an operative risk factor for PPH, analysis of the effect of duration of surgery and incision delivery interval on intraoperative blood loss using multiple linear regression showed that these two variables only accounted for 19.1% of the intraoperative blood loss. The decrease in the effect of the longer duration of surgery on the blood loss in the treatment arm compared with the placebo arm was probably due to the effects of sustained contractility and effective blood coagulation induced by misoprostol and TXA, respectively. Usually, the longer the duration of surgery, the greater the intraoperative blood loss. To minimise the effects of other cofounders on the outcome, women recruited into the research had similar demographic and obstetric risk profiles in both groups. Also, the surgeries were performed by experienced specialist doctors, and the same method of anaesthesia (sub-arachnoid block) and surgical techniques were adopted for all the participants.

Strength and the limitations of the study

The study examined the benefits of the combined effect of misoprostol and tranexamic acid on intraoperative blood loss during caesarean sections. Most of the studies reviewed focused on each drug individually.

The study result was potentially limited by the exclusion of high-risk pregnancies; these women have a higher risk for PPH and would have potentially benefited from the study. Thus, future studies could incorporate these sets of women.

## Conclusions

Preoperative administration of 600 mcg of sublingual misoprostol and 1000 mg of intravenous tranexamic acid is associated with a statistically significant reduction in the amount of intraoperative blood loss during caesarean section. This regimen also lowers the risk of postpartum anaemia, the use of extra uterotonics, and blood transfusion in women undergoing caesarean delivery.
